# Supplementary material for: Dysbacteriosis-Derived Lipopolysaccharide Causes Embryonic Osteopenia through Retinoic-Acid-Regulated DLX5 Expression
Source: Int J Mol Sci. 2020 Apr 4;21(7):2518. doi: 10.3390/ijms21072518 (PMC7177785; doi:10.3390/ijms21072518)
Supplement: Supplementary file 1 [file ijms-21-02518-s001.docx]

**
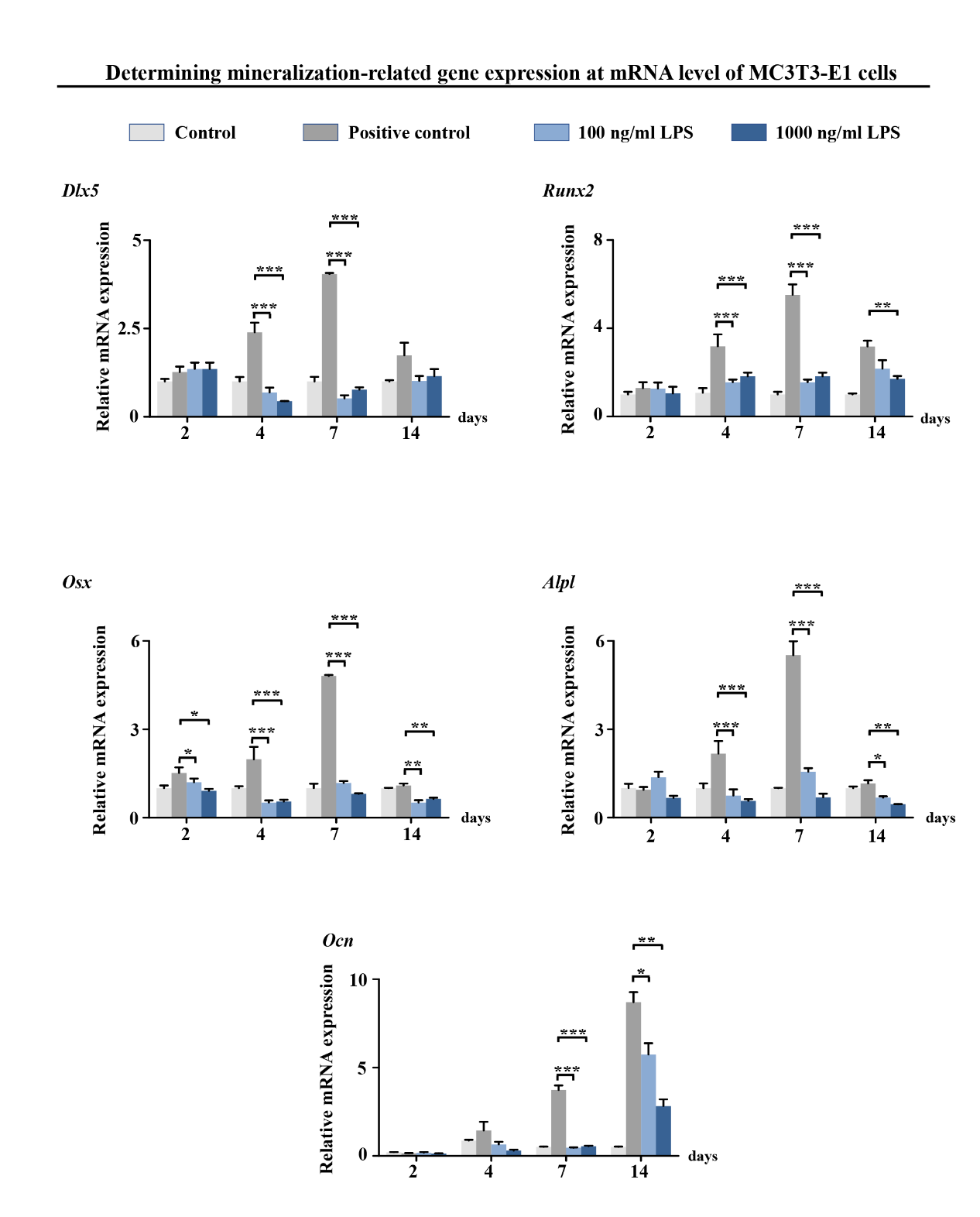
Supplementary material**

**Figure S1.** Determining osteogenesis-related gene expression at mRNA level in MC3T3-E1 cells. qPCR data showing the mRNA expression of *Dlx5*, *Runx2*, *Osx*, *Alpl*, and *Ocn* in MC3T3-E1 cells, which were cultured for 2, 4, 7, and 14 days from control, positive (inducing) control, 100 ng/mL LPS-treated, and 1000 ng/mL LPS-treated groups. The control cells were cultured in normal complete medium. Positive control cells were cultured with osteogenic differentiation medium. LPS-treated cells were cultured with osteogenic differentiation medium supplemented with 100 ng/ml or 1000 ng/ml LPS.

***
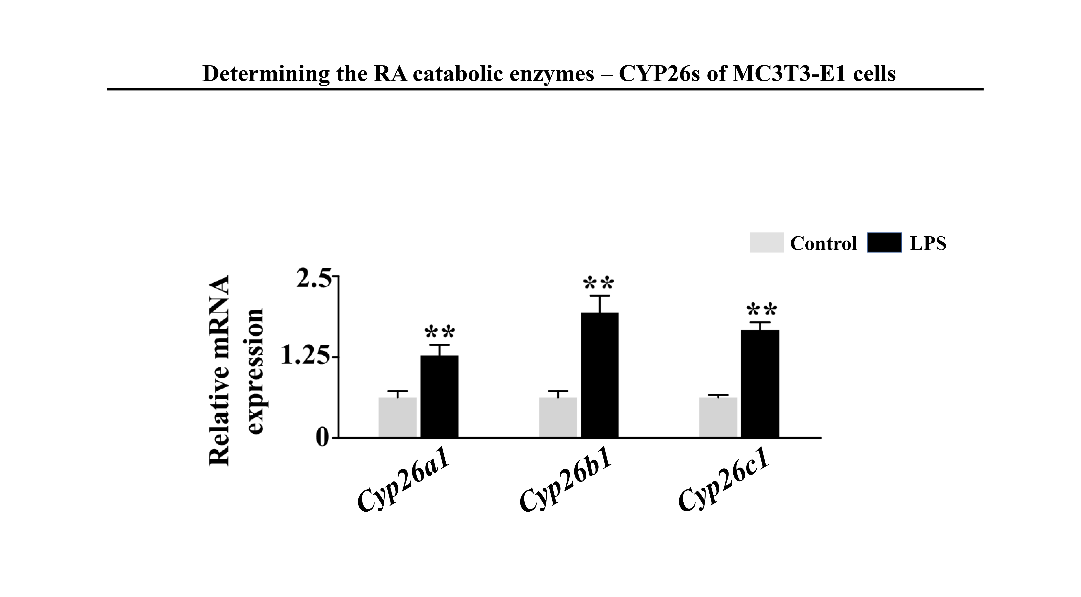
***


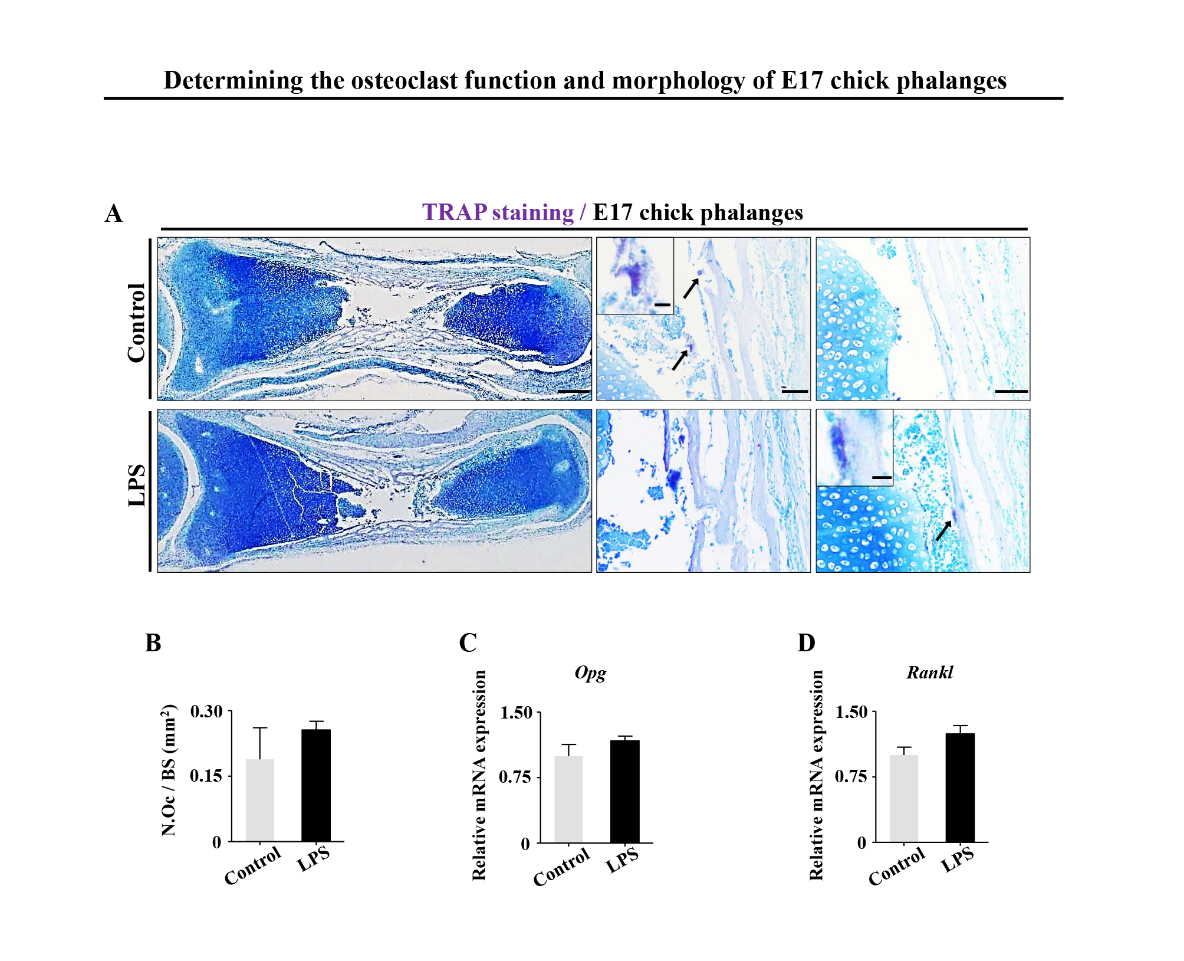
**Figure S2.** The assessment of RA catabolic enzymes - CYP26s genes expression in MC3T3-E1 cells. qPCR data showing the mRNA expression of *Cyp26a1*, *Cyp26b1* and *Cyp26c1* in control and LPS-treated groups.

**Figure S3. Determining the osteoclasts morphology and function in E17 chick phalanges.** (A) Representative images of tartrate-resistant acid phosphatase (TRAP) staining on longitudinal sections of E17 chick phalanges from control and LPS-treated groups. The right panels are the higher magnification images. (**B**) Bar charts showing the ratios of number of osteoclasts to bone surface between control and LPS-treated groups. (**C**-**D**) qPCR data showing the mRNA expression of *Opg* (**C**) and *Rankl* (**D**) in control and LPS-treated groups. Scale bars = 300 μm in left panels; 10 μm in top right panels; 66 μm in lower right panels.


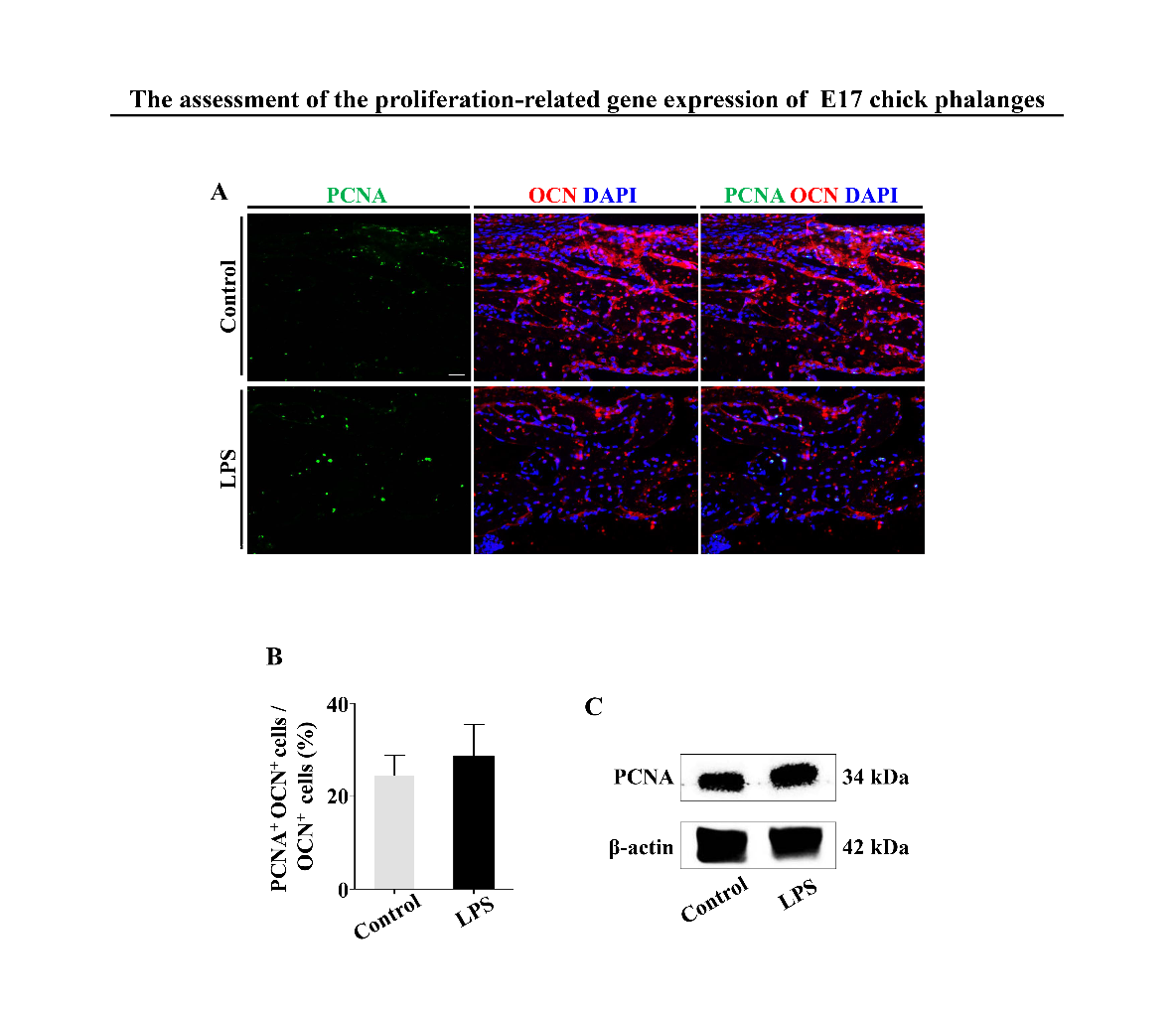


**Figure S4.** **The assessment of the proliferation-related gene expression in E17 chick phalanges.** (**A**) Representative immunofluorescent staining of PCNA and OCN on the longitudinal sections of E17 chick phalanges trabecular bone from control and LPS-treated groups, counterstained with DAPI. (**B**) Bar chart showing the ratio of PCNA and OCN immunofluorescent double staining positive cells to total OCN positive cells. (**C**) Western blot showing the expression of PCNA from control and LPS-treated groups. Scale bars = 50 μm in A.


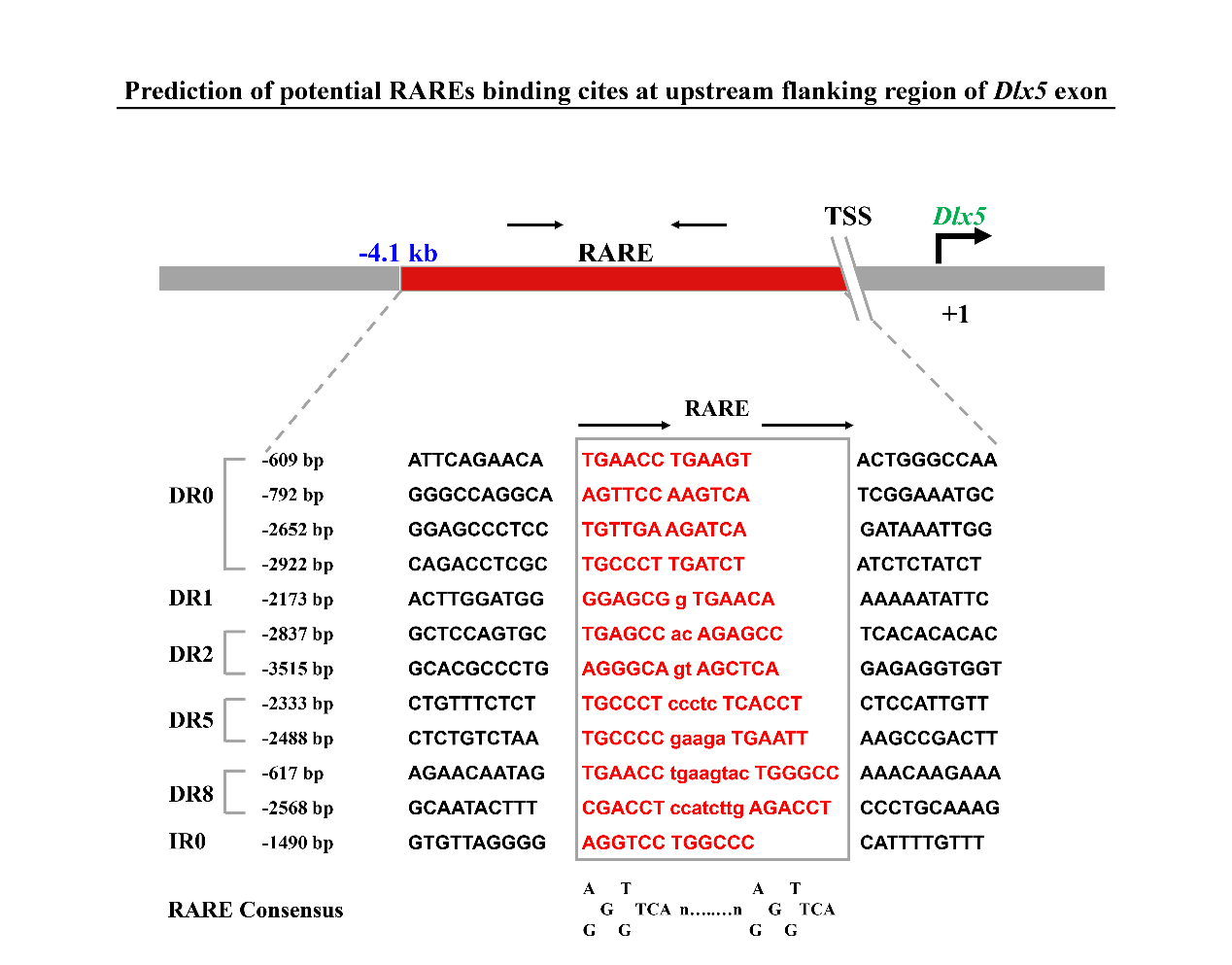


**Figure S5. Prediction of potential RAREs binding cites at upstream flanking region of the exon of *Dlx5* in MC3T3-E1 cells.** In silico analysis of the ∼4.1-kb mouse *Dlx5* promoter region, using NUBIScan, identified multiple potential RAREs.

**
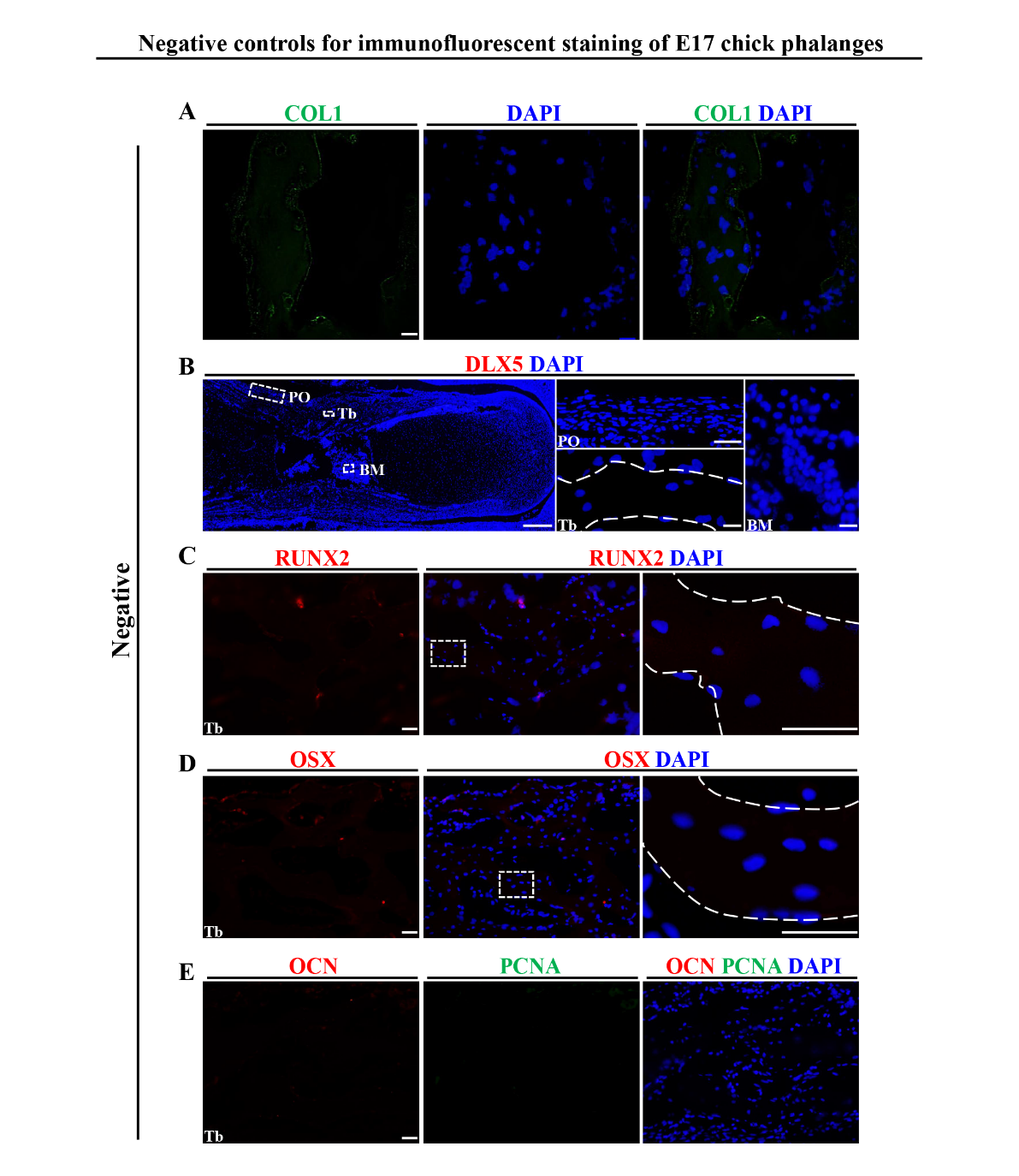
**

**Figure S6.** Negative controls of E17 chick phalanges for COL1, DLX5, RUNX2, OSX, OCN and PCNA immunofluorescent staining. Scale bars = 10 μm in A; 50 μm in B-E.


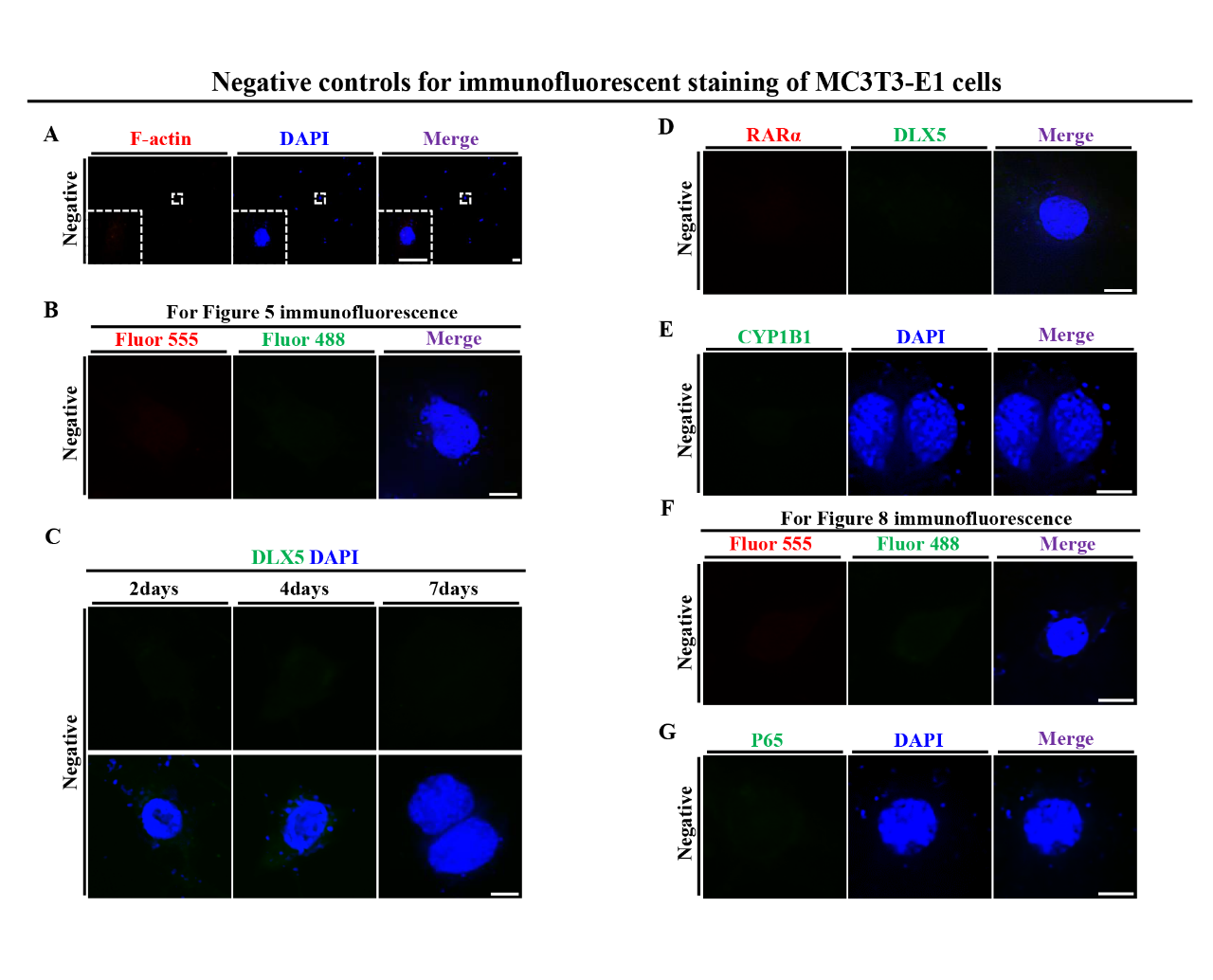


**Figure S7.** Negative controls of MC3T3-E1 cells for F-actin (A, Figure 4), DLX5 (C, Figure 6), RARα, DLX5 and CYP1B1 (D-E, Figure 7), P65 (G, Figure 9) immunofluorescent staining. B and F are 2^nd^ antibody staining controls for Figure 5 (B) and Figure 8 (F), respectively. Scale bars = 50 μm in A; 20 μm in B-G.


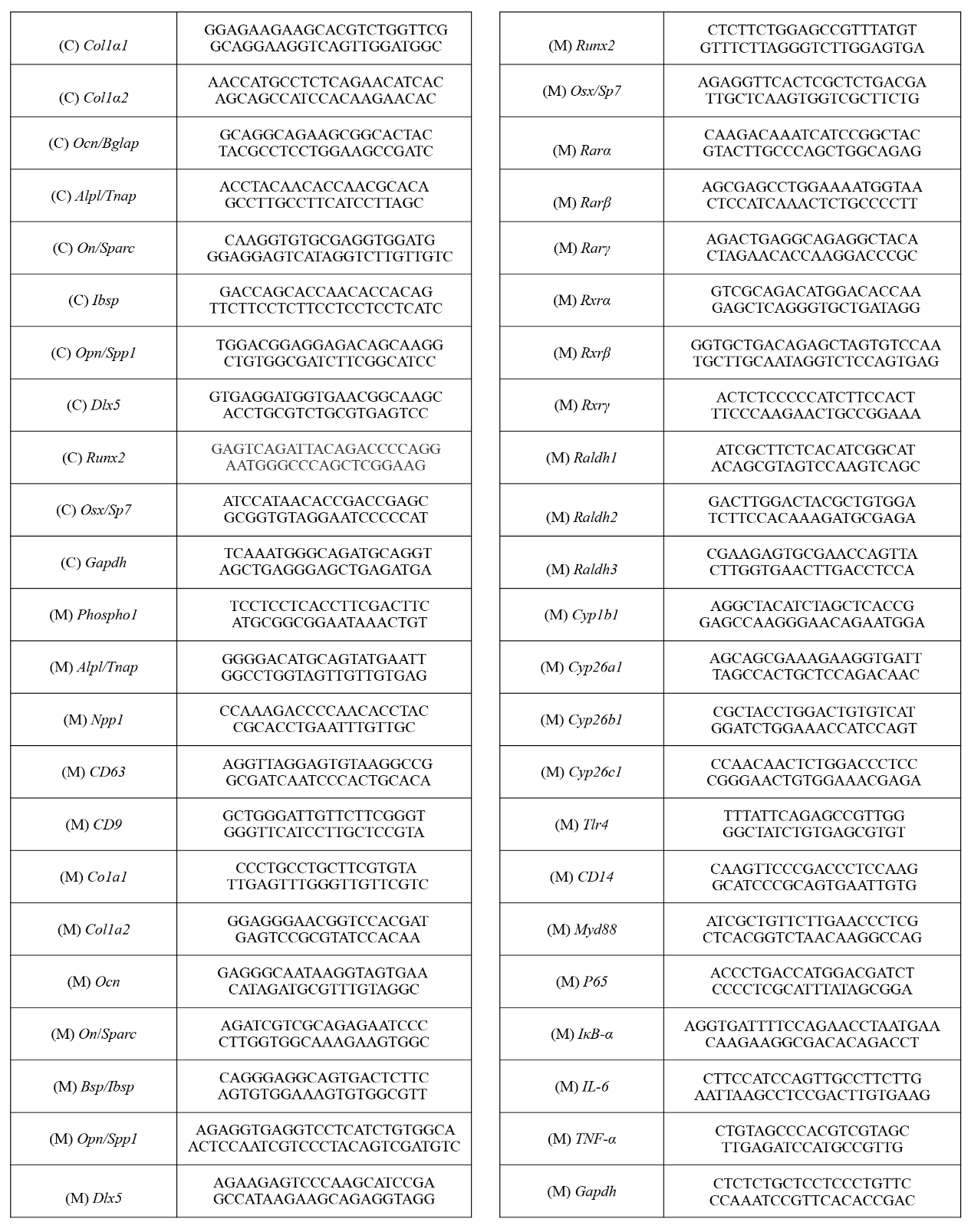


**Table S1. The sets of primers used in this study.**


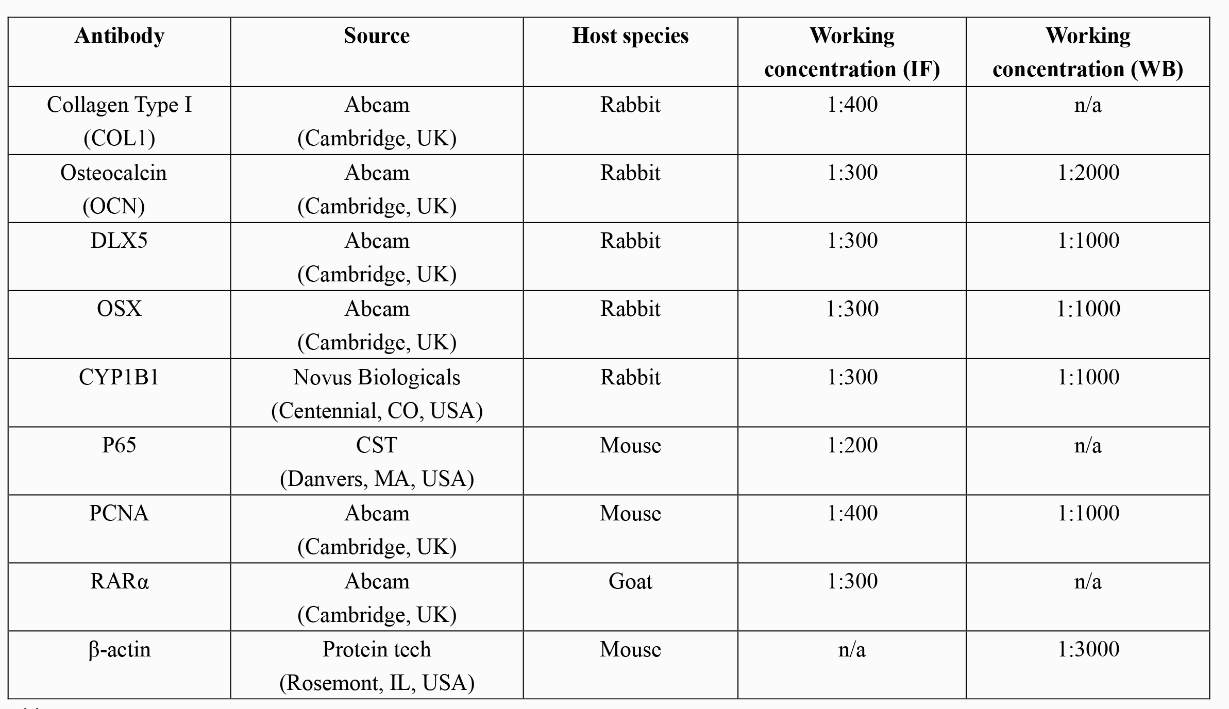


**Table S2: List of primary antibodies, the source, host species and working dilutions.** IF: Immunofluorescence; WB: Western blot.
